# Metabolic traits as mediators between excess salt intake and the risk of nonalcoholic fatty liver disease

**DOI:** 10.1097/MD.0000000000042715

**Published:** 2025-07-18

**Authors:** Rui Li, Kaitai Hu, Lei Cui, Shiyi Sun, Yinghai Xie

**Affiliations:** a The First Affiliated Hospital of Anhui University of Science and Technology, Huainan, Anhui Province, People’s Republic of China; b The First Affiliated Hospital of Nanjing Medical University, Nanjing, Jiangsu Province, People’s Republic of China.

**Keywords:** acetate levels, Mendelian randomization, metabolic traits, nonalcoholic fatty liver disease, salt added to food

## Abstract

Nonalcoholic fatty liver disease (NAFLD) affects approximately 25% of individuals worldwide and poses a significant burden on global health. In this study, we conducted Mendelian randomization analysis using salt added to food (SAF) as the exposure and investigated 249 metabolic traits as potential mediating factors. We applied multiple methods, including inverse-variance-weighted, weighted median, and Mendelian randomization-Egger, to assess causal relationships and detect pleiotropy. Mediation analysis was conducted to identify potential mediators between SAF and NAFLD. Our analysis revealed that higher SAF is associated with an increased risk of NAFLD (beta = 0.38, *P* = .0056). We identified 6 metabolites associated with SAF. Additionally, we found 106 metabolites associated with NAFLD. Acetate levels were identified as the primary mediator between SAF and NAFLD, mediating approximately 28.08% of the effect. Our study elucidates potential mechanisms by which excessive salt intake increases the risk of NAFLD, highlighting the role of acetate levels as a key mediator. These findings contribute to our understanding of the relationship between salt intake and NAFLD and may inform strategies for prevention and treatment. However, further research is warranted to validate these findings and explore additional mediators and mechanisms involved.

## 1. Introduction

About 25% of individuals worldwide are estimated to be afflicted with nonalcoholic fatty liver disease (NAFLD), which is the most prevalent form of chronic liver disease.^[1]^ NAFLD may develop into cirrhosis, liver fibrosis, and eventually hepatocellular cancer.^[2]^ Therefore, NAFLD imposes a significant burden on global health.^[3]^ NAFLD not only increases the disease burden on individuals but also adds to the burden of healthcare costs and resources, posing a significant global health challenge.^[4]^

The mechanism underlying the occurrence of NAFLD remains unclear at present. Previous studies^[5–8]^ have reported that higher salt intake is associated with an increased risk of NAFLD. A prospective study^[5]^ from China involving 35,023 participants found an association between higher salt intake and increased risk of NAFLD after an 8-year follow-up. A cross-sectional study^[6]^ from Korea involving 27,433 participants found an association between higher sodium intake and increased risk of NAFLD. Another cross-sectional study^[7]^ targeting the American population also identified an association between higher sodium intake and increased risk of NAFLD. A meta-analysis^[8]^ of 7 studies also yielded a similar conclusion. However, the mechanisms by which excessive salt intake increases the risk of NAFLD are not yet fully understood.

Therefore, we conducted this Mendelian randomization (MR) study, using salt added to food (SAF) as the exposure, and investigating 249 metabolic traits as potential mediating factors to explore the underlying mechanisms by which SAF affects NAFLD. MR is a statistical method employed in observational studies that utilizes genetic variants as instrumental variables (IVs) to evaluate causal relationships between various exposures and outcomes.^[9]^ The aim of this study is to identify potential metabolic mediators and gain insights into the causal pathway linking SAF to NAFLD.

## 2. Methods

### 2.1. Overview

**Figure 1.**
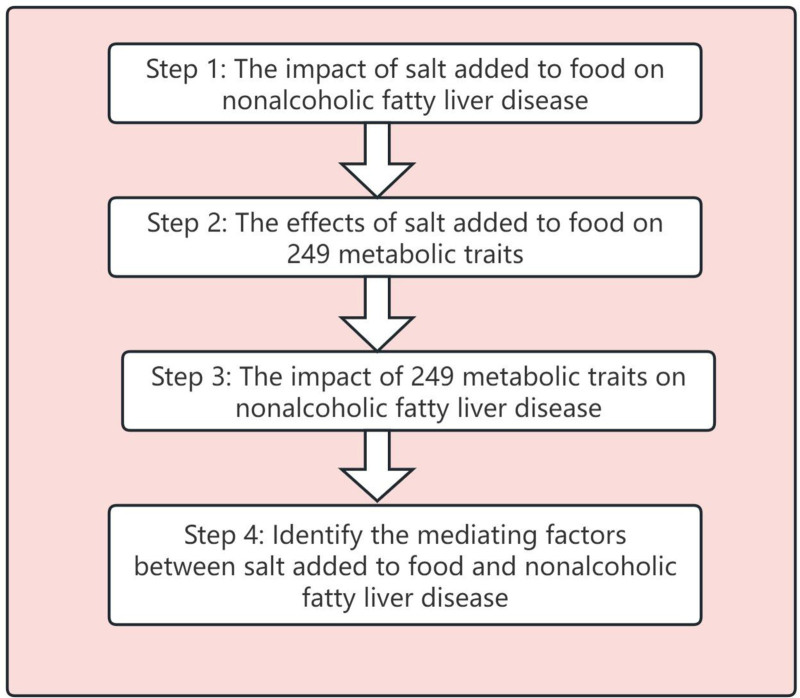
Analysis process flowchart. This figure outlines our analytical process, which consists of 4 key steps: First, we examined the association between dietary salt intake and nonalcoholic fatty liver disease (NAFLD) (Step 1). Next, we investigated the effects of dietary salt intake on 249 metabolic traits (Step 2), followed by an assessment of the relationship between these metabolic traits and NAFLD (Step 3). Finally, we integrated these findings to identify potential mediating metabolic traits linking dietary salt intake to NAFLD (Step 4).

The MR analysis is based on the following premises: the impact of IVs on the outcome is solely mediated through the exposure variable. IVs are assumed to affect exposure exclusively, with no other mechanisms influencing the outcome; there exists no direct association between IVs and the outcome; IVs indirectly influence the outcome solely through their effects on the exposure. As shown in Figure 1, the analytical procedure for this investigation may be broadly broken down into 4 steps. First, we analyzed the impact of SAF on NAFLD (Step 1). Second, we investigated the effects of SAF on 249 metabolic traits (Step 2). Third, we assessed the impact of 249 metabolic traits on NAFLD (Step 3). Finally, we integrated the impact of SAF on 249 metabolic traits with the effect of 249 metabolic traits on NAFLD to identify the mediating factors between SAF and NAFLD (Step 4).

### 2.2. Data sources

The OpenGWAS project^[10]^ (https://gwas.mrcieu.ac.uk/) is the primary source of the summary-level genome-wide association study (GWAS) data utilized in this investigation. As shown in Table 1, we utilized SAF as the exposure, 249 metabolic traits as potential mediators, and NAFLD as the outcome. The OpenGWAS project^[10]^ extracted the summary-level GWAS of SAF (sample size: 462,630) used as the exposure from the UK Biobank. The OpenGWAS project collected summary-level GWAS data on the 249 metabolic traits (sample size ranges from 110,051 to 115,082) utilized as potential mediators in this study from a published article.^[11]^ A meta-analysis^[12]^ provided the summary-level GWAS data of NAFLD (8434 cases and 770,180 controls) that was utilized as the outcome. This MR analysis conducted in this study utilized data obtained from publicly available databases. Therefore, this research was exempt from review by an ethics committee.

**Table 1:** Information of the outcome, mediating factors, and exposures.

	Name	IEU OpenGWAS project ID	Sample size	Data source	Number of cases	Number of controls
Exposure	Salt added to food	ukb-b-8121	462,630	The UK Biobank	NA	NA
Mediating factors	249 metabolic traits	ebi-a-GCST90092803–ebi-a-GCST90093051	110,051–115,082	PMID:35213538	NA	NA
Outcome	Nonalcoholic fatty liver disease	ebi-a-GCST90091033	778,614	PMID:34841290	8434	770,180

The presence of salt added to food was assessed using the ACE touchscreen questionnaire item: “Do you add salt to your food? (Exclude salt used in cooking).” Respondents were provided with the following response options: 1. Never/Rarely, 2. Sometimes, 3. Usually, and 4. Always. Additional information regarding salt added to food can be obtained from the UK Biobank website (https://biobank.ctsu.ox.ac.uk/crystal/field.cgi?id=1478). More information about exposure, mediating factors, and outcome is available at the IEU OpenGWAS project (https://gwas.mrcieu.ac.uk/).

GWAS = genome-wide association studies, IEU = Integrative Epidemiology Unit, NA = not applicable.

### 2.3. The IVs selection

In MR investigation, single nucleotide polymorphisms (SNPs) have been employed as IVs to examine the causal connection between exposure factors and outcomes. The process of SNP selection involved specific criteria: low linkage disequilibrium (*r*^2^ < 0.001), an *F*-statistic exceeding 10, a clumping window larger than 10,000 kb, and significance below the genome-wide threshold (*P* < 5 × 10^–8^). To prevent unnecessary bias, we excluded SNPs significantly (*P* < 5 × 10^–8^) linked to the outcome in each analysis.

### 2.4. Statistical analysis

To identify potential causal linkages, the inverse-variance-weighted (IVW) method served as the primary approach, complemented by the utilization of the weighted median and MR-Egger methods in this investigation. The IVW technique, which possesses the strongest capacity for determining causal linkages, necessitates either balanced or negligible horizontal pleiotropy.^[13]^ The MR-Egger approach can be used to measure horizontal pleiotropy as it permits nonzero intercepts. We employ false discovery rate calibration^[14]^ to control the quantity of false positives generated during multiple hypothesis testing. For evaluating heterogeneity, Cochran *Q*-test was conducted. The fixed-effects IVW model was utilized when heterogeneity was absent (*P* > .05), and the random-effects IVW model was employed when heterogeneity was present (*P* < .05). We employed the MR-Presso technique to detect any outliers. It is noteworthy that the MR-Presso technique is also a causal inference method, allowing for direct estimation of results after outlier removal. Using R (version 4.3.2, R Foundation for Statistical Computing, Vienna, Austria) and the TwoSampleMR package^[15]^ (version 0.5.10, Bristol Medical School (University of Bristol), Bristol, United Kingdom), we conducted all statistical analyses.

### 2.5. Mediation analysis

In this study, we conducted a 2-step mediation MR analysis employing 249 metabolites as potential mediators. In Step 1, we analyzed the effect of SAF on the potential mediator (β1). In Step 2, we examined the impact of the potential mediator on NAFLD (β2). By calculating the product of β1 and β2, the size of the mediation effect can be obtained. Subsequently, we calculated the magnitude of the mediation effect (β1 × β2), followed by the calculation of the proportion of the total effect mediated by the mediator.

## 3. Results

This study employed the mediation MR method to investigate the mechanisms by which SAF increases the risk of NAFLD.

### 3.1. The impact of SAF on NAFLD

As shown in Table S1, Supplemental Digital Content 1, https://links.lww.com/MD/P265, our analysis revealed that higher SAF increases the risk of NAFLD (beta = 0.38, *P* = .0056).

### 3.2. The impact of SAF on potential mediators

**Figure 2.**
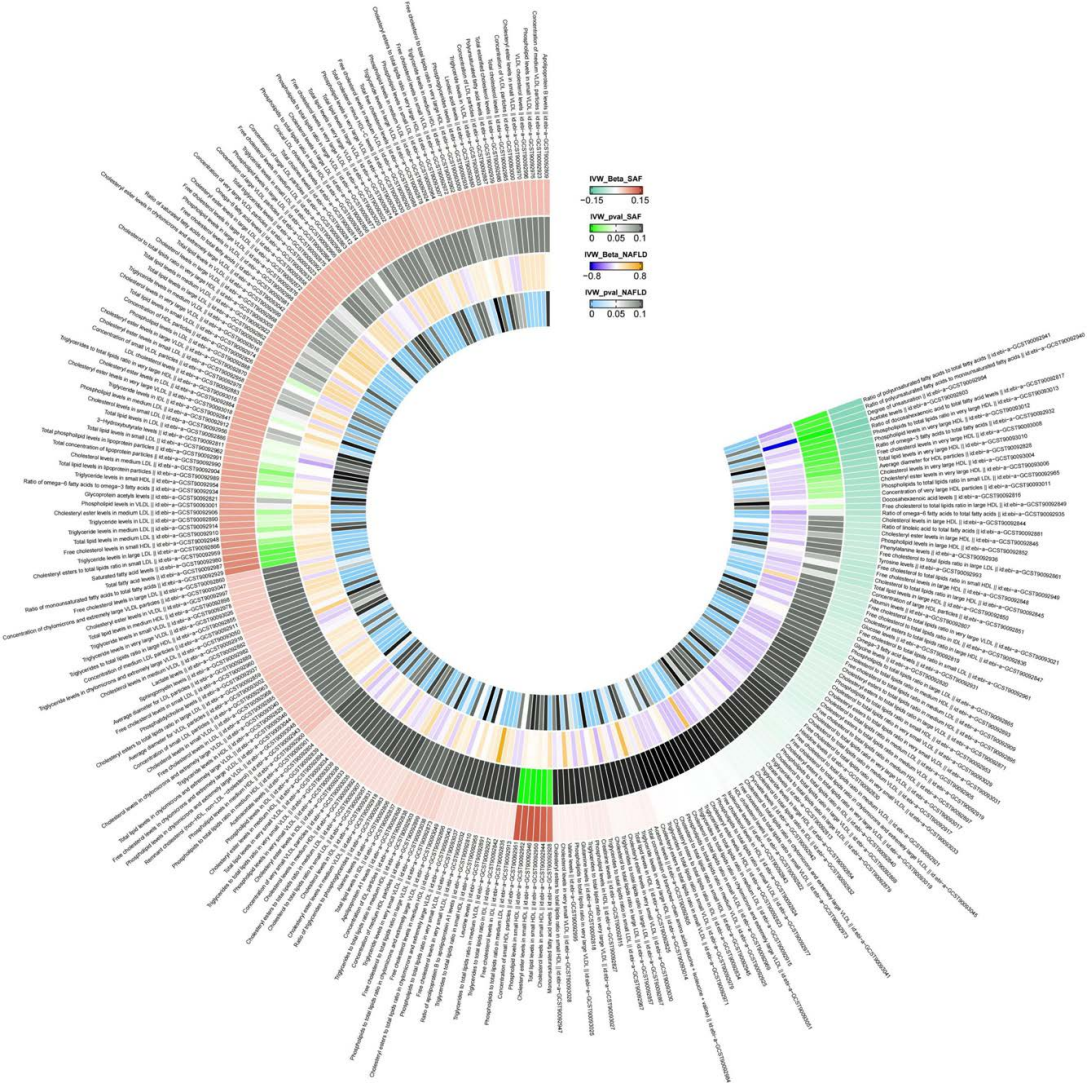
The effects of salt added to food on 249 metabolic traits and the effects of 249 metabolic traits on nonalcoholic fatty liver disease. We visualized the effects of salt added to food (SAF) on 249 metabolic traits and their subsequent associations with nonalcoholic fatty liver disease (NAFLD) using a circular heatmap. The outermost ring displays the 249 metabolic traits along with their IDs, followed by two concentric rings representing the beta coefficients and *P*-values for SAF’s effects on these traits. Moving inward, the next two rings show the beta coefficients and *P*-values for the associations between these metabolic traits and NAFLD, with color gradients indicating effect sizes and statistical significance as depicted in the legend. IVW = inverse-variance-weighted method, NAFLD = nonalcoholic fatty liver disease, SAF = salt added to food.

We separately analyzed the effects of SAF on 249 metabolites and presented the results in Table S2, Supplemental Digital Content 2, https://links.lww.com/MD/P266. Additionally, we visualized these results in Figure 2. Subsequently, we identified metabolites associated with SAF and free from horizontal pleiotropy through *q*-value (*P*-value post-false discovery rate corrected). Finally, we identified 6 metabolites associated with SAF, namely acetate levels (beta = −0.13, *P* = .00054), cholesterol levels in small high-density lipoprotein (HDL) (beta = 0.14, *P* = .00036), cholesteryl ester levels in small HDL (beta = 0.15, *P* = .00016), total lipid levels in small HDL (beta = 0.16, *P* = .00030), concentration of small HDL particles (beta = 0.15, *P* = .00027), and phospholipid levels in small HDL (beta = 0.15, *P* = .00068). Additional information about these analysis results is provided in Table S3, Supplemental Digital Content 3, https://links.lww.com/MD/P267.

### 3.3. The impact of potential mediators on NAFLD

We analyzed the effects of 249 metabolites on NAFLD and presented all analysis results in Table S4, Supplemental Digital Content 4, https://links.lww.com/MD/P268. We also visualized these results in Figure 2. Subsequently, we identified metabolites associated with NAFLD and free from horizontal pleiotropy through *q*-value. As depicted in Table S5, Supplemental Digital Content 5, https://links.lww.com/MD/P269, we identified 106 metabolites associated with NAFLD through screening.

### 3.4. Metabolites mediate the association between SAF and the risk of NAFLD

As shown in Table 2, we identified potential mediators between SAF and NAFLD, including acetate levels, cholesterol levels in very large HDL, cholesteryl ester levels in very large HDL, cholesteryl esters to total lipids ratio in small low-density lipoprotein, free cholesterol levels in very large HDL, phospholipids to total lipids ratio in small low-density lipoprotein, ratio of monounsaturated fatty acids to total fatty acids, and total fatty acid levels, based on *P*-values. However, when employing *q*-values for screening, only acetate levels remain as a mediator. Among these, we found that acetate is the primary metabolite mediating the relationship between SAF and NAFLD, with acetate mediating approximately 28.08% of the effect of SAF increasing the risk of NAFLD. The proportion of the total effect mediated by the mediation effects of the other 7 metabolites ranges from approximately 3.42% to 5.75%. For more information, please refer to Table 2.

**Table 2:** The metabolic traits mediating between salt added to food (SAF) and nonalcoholic fatty liver disease.

The impact of salt added to food on mediators										The impact of mediators on nonalcoholic fatty liver disease											The proportion of the total effect mediated by this mediator (%)
Outcome	Exposure	Number of SNPs	IVW_Beta	IVW_se	IVW_pval	IVW_q_value	Egger_intercept	se	pval	Outcome	Exposure	Number of SNPs	IVW_Beta	IVW_se	IVW_pval	IVW_q_value	Egger_intercept	se	pval	β1 × β2	
Acetate levels ‖ id:ebi-a-GCST90092803	Salt added to food ‖ id:ukb-b-8121	102	−1.28E−01	3.70E−02	5.38E−04	2.68E−02	7.61E−05	1.74E−03	9.65E−01	Nonalcoholic fatty liver disease ‖ id:ebi-a-GCST90091033	Acetate levels ‖ id:ebi-a-GCST90092803	5	−8.39E−01	3.51E−01	1.69E−02	3.78E−02	−2.32E−02	4.07E−02	6.08E−01	1.07E−01	28.08
Cholesterol levels in very large HDL ‖ id:ebi-a-GCST90093004	Salt added to food ‖ id:ukb-b-8121	100	−9.20E−02	3.96E−02	2.00E−02	2.16E−01	−2.86E−03	1.84E−03	1.24E−01	Nonalcoholic fatty liver disease ‖ id:ebi-a-GCST90091033	Cholesterol levels in very large HDL ‖ id:ebi-a-GCST90093004	66	−1.42E−01	5.98E−02	1.73E−02	3.85E−02	−3.69E−03	5.28E−03	4.87E−01	1.31E−02	3.43
Cholesteryl ester levels in very large HDL ‖ id:ebi-a-GCST90093006	Salt added to food ‖ id:ukb-b-8121	100	−8.73E−02	3.95E−02	2.70E−02	2.24E−01	−3.03E−03	1.83E−03	1.01E−01	Nonalcoholic fatty liver disease ‖ id:ebi-a-GCST90091033	Cholesteryl ester levels in very large HDL ‖ id:ebi-a-GCST90093006	71	−1.53E−01	6.03E−02	1.12E−02	2.77E−02	−4.92E−03	5.10E−03	3.38E−01	1.33E−02	3.49
Cholesteryl esters to total lipids ratio in small LDL ‖ id:ebi-a-GCST90092959	Salt added to food ‖ id:ukb-b-8121	100	9.35E−02	3.75E−02	1.27E−02	1.50E−01	2.20E−03	1.75E−03	2.13E−01	Nonalcoholic fatty liver disease ‖ id:ebi-a-GCST90091033	Cholesteryl esters to total lipids ratio in small LDL ‖ id:ebi-a-GCST90092959	34	1.63E−01	6.13E−02	7.70E−03	2.11E−02	−3.57E−03	7.75E−03	6.49E−01	1.53E−02	4.00
Free cholesterol levels in very large HDL ‖ id:ebi-a-GCST90093008	Salt added to food ‖ id:ukb-b-8121	100	−1.07E−01	4.00E−02	7.54E−03	1.12E−01	−2.24E−03	1.87E−03	2.33E−01	Nonalcoholic fatty liver disease ‖ id:ebi-a-GCST90091033	Free cholesterol levels in very large HDL ‖ id:ebi-a-GCST90093008	55	−1.49E−01	6.54E−02	2.29E−02	4.82E−02	−3.38E−03	5.89E−03	5.69E−01	1.59E−02	4.16
Phospholipids to total lipids ratio in small LDL ‖ id:ebi-a-GCST90092965	Salt added to food ‖ id:ukb-b-8121	100	−8.62E−02	3.82E−02	2.39E−02	2.21E−01	−1.79E−03	1.79E−03	3.20E−01	Nonalcoholic fatty liver disease ‖ id:ebi-a-GCST90091033	Phospholipids to total lipids ratio in small LDL ‖ id:ebi-a-GCST90092965	34	−1.85E−01	6.44E−02	3.98E−03	1.23E−02	8.55E−04	8.09E−03	9.17E−01	1.60E−02	4.18
Ratio of monounsaturated fatty acids to total fatty acids ‖ id:ebi-a-GCST90092929	Salt added to food ‖ id:ukb-b-8121	101	1.14E−01	4.29E−02	7.83E−03	1.12E−01	3.79E−03	1.99E−03	5.93E−02	Nonalcoholic fatty liver disease ‖ id:ebi-a-GCST90091033	Ratio of monounsaturated fatty acids to total fatty acids ‖ id:ebi-a-GCST90092929	46	1.68E−01	6.25E−02	7.15E−03	2.00E−02	2.17E−03	6.28E−03	7.31E−01	1.92E−02	5.02
Total fatty acid levels ‖ id:ebi-a-GCST90092987	Salt added to food ‖ id:ukb-b-8121	100	1.05E−01	4.00E−02	8.57E−03	1.12E−01	3.33E−03	1.85E−03	7.53E−02	Nonalcoholic fatty liver disease ‖ id:ebi-a-GCST90091033	Total fatty acid levels ‖ id:ebi-a-GCST90092987	50	2.09E−01	7.26E−02	3.99E−03	1.23E−02	6.26E−03	7.37E−03	4.00E−01	2.20E−02	5.75

The metabolic traits mediating between salt added to food (SAF) and nonalcoholic fatty liver disease were screened based on the IVW_p_value (<0.05), and those with horizontal pleiotropy were excluded. The left part of the table displays the effect of SAF on potential mediators (IVW method) and the results of horizontal pleiotropy. The right part of the table shows the effect of potential mediators on nonalcoholic fatty liver disease and the results of horizontal pleiotropy.The MR-Egger method is employed for detecting horizontal pleiotropy due to its allowance for the presence of nonzero intercepts (the columns in the table corresponding to “egger_intercept,” “se,” and “pval”), and a pval below 0.05 suggests the existence of horizontal pleiotropy. β1 × β2: The mediation effect is calculated as the product of the beta coefficient representing the effect of SAF on the metabolite and the beta coefficient representing the effect of this metabolite on NAFLD. The proportion of the total effect mediated by the mediator is calculated by dividing β1 × β2 by the beta coefficient of the effect of salt added to food on nonalcoholic fatty liver disease, using the IVW method.

HDL = high-density lipoprotein, IVW = inverse-variance-weighted, LDL = low-density lipoprotein, NAFLD = nonalcoholic fatty liver disease.

## 4. Discussion

This study utilized MR analysis to investigate the impact of SAF on NAFLD and aimed to elucidate its underlying mechanisms. The findings of our study can be summarized as follows: Firstly, excessive salt intake increases the risk of NAFLD. Secondly, excessive salt intake may influence the metabolism of certain metabolites, thereby affecting their levels. Thirdly, there is a very close relationship between metabolites and NAFLD, with over 100 metabolites found to be associated with NAFLD. Lastly, excessive salt intake can increase the risk of NAFLD by influencing the levels of metabolites such as acetate. It is noteworthy that in some of our analyses, we deemed them invalid due to the presence of horizontal pleiotropy. Consequently, certain potential mediators were not identified.

Previous studies^[5–8]^ have reported that excessive salt intake increases the risk of NAFLD. A prospective study^[5]^ from China involving 35,023 participants found an association between higher salt intake and increased risk of NAFLD after an 8-year follow-up. A cross-sectional study^[6]^ from Korea involving 27,433 participants found an association between higher sodium intake and increased risk of NAFLD. Another cross-sectional study^[7]^ targeting the American population also identified an association between higher sodium intake and increased risk of NAFLD. A meta-analysis^[8]^ of 7 studies also yielded a similar conclusion. Overall, current researches support an association between higher salt intake and increased risk of NAFLD. This is consistent with the findings of our study, where we observed a significant increase in the risk of NAFLD with the frequency of SAF.

The mechanism underlying the association between high salt intake and NAFLD remains unclear. Currently, it is generally believed that high salt intake may affect NAFLD by influencing insulin resistance, endogenous fructose metabolism, and the function of the renin-angiotensin-aldosterone system (RAAS). High salt intake may lead to insulin resistance,^[16–18]^ which is closely associated with NAFLD.^[19]^ A study^[20]^ found that high salt intake increases endogenous fructose levels, leading to fatty liver. High salt intake inhibits the RAAS.^[21]^ Evidence from rat models indicates that inhibiting the RAAS pathway may lessen oxidative stress on the hepatocyte, which lowers the risk of NAFLD.^[22,23]^ Although the mechanisms mentioned above may exist, the exact mechanisms by which excessive salt intake increases the risk of NAFLD remain incompletely elucidated. Dietary intake is absorbed by the intestines and transported through the bloodstream, implying that substances in the blood may serve as crucial intermediaries linking salt intake to NAFLD. Thus, we utilized metabolites in the blood as mediators and conducted this MR analysis to identify potential mechanisms underlying the association between excessive salt intake and NAFLD. Through our analysis, we found that the most important metabolite mediating the relationship between higher salt intake and NAFLD is acetate levels, mediating approximately 28.08% of this risk. A study^[24]^ on mice found that a high-salt diet can increase acetate levels in feces by affecting gut microbiota. However, we did not find any studies investigating the relationship between high salt intake and acetate levels in the blood. Based on our study, we hypothesize that excessive salt intake may inhibit the absorption of acetate levels or promote their excretion, leading to an increase in acetate levels in feces and a decrease in acetate levels in the blood. A study^[25]^ in mice found that increasing acetate intake can inhibit the progression of NAFLD. Our study found that higher salt intake reduces acetate levels in the blood, while higher acetate levels can decrease the risk of NAFLD. Our research extends the findings of these 2 previous studies, indicating that the increased risk of NAFLD associated with excessive salt intake is largely mediated by acetate levels in the blood.

Although our study elucidates the potential mechanisms by which excessive salt intake increases the risk of NAFLD, caution must be exercised when interpreting these research findings. The results of the MR study suggested that long-term exposure to high salt consumption can cause NAFLD, meaning that immediate effects might not be clinically significant.

Undoubtedly, this study inevitably possesses inherent limitations. Firstly, our study was confined to qualitative analysis rather than quantitative analysis. This constraint stemmed from the method of assessing higher salt intake, which focused on determining whether additional salt was frequently added to food, thereby limiting more nuanced analyses. Secondly, as the exposure, mediating factors, and outcome data were predominantly gathered from European populations, the generalizability of our findings to other demographic groups remains uncertain. Thirdly, another significant limitation of our study is our inability to perform gender- and age-stratified analyses due to the absence of summary-level GWAS data stratified by these variables. Given the intricate interplay between gender, age, and NAFLD, a deeper exploration of these subpopulations could offer valuable insights into the differential effects of salt intake on NAFLD. However, without access to stratified data, we are precluded from elucidating such nuances, underscoring the imperative for future research endeavors to address this gap.

## 5. Conclusion

This study found the following: excessive salt intake affects the levels of metabolites in the blood; excessive salt intake increases the risk of NAFLD; there is a close correlation between metabolites in the blood and NAFLD; excessive salt intake can increase the risk of NAFLD by affecting the levels of certain metabolites in the blood, such as acetate.

## Acknowledgments

The authors thank the UK Biobank, and the IEU OpenGWAS project developed by The MRC Integrative Epidemiology Unit (IEU) at the University of Bristol. We thank Dr. Wenwen Yang and Dr. Hongzhen Zhang for their technical assistance in data processing and manuscript editing.

## Author contributions

**Conceptualization:** Rui Li, Kaitai Hu, Lei Cui, Shiyi Sun, Yinghai Xie.

**Data curation:** Rui Li, Yinghai Xie.

**Formal analysis:** Rui Li, Shiyi Sun, Yinghai Xie.

**Funding acquisition:** Rui Li, Shiyi Sun, Yinghai Xie.

**Investigation:** Rui Li, Kaitai Hu, Yinghai Xie.

**Methodology:** Rui Li, Kaitai Hu, Yinghai Xie.

**Project administration:** Rui Li, Kaitai Hu, Yinghai Xie.

**Resources:** Rui Li, Yinghai Xie.

**Software:** Rui Li, Yinghai Xie.

**Supervision:** Rui Li, Yinghai Xie.

**Validation:** Rui Li, Lei Cui, Yinghai Xie.

**Visualization:** Rui Li, Yinghai Xie.

**Writing – original draft:** Rui Li, Yinghai Xie.

## Supplementary Material


